# The way forward for integrated community case management programmes: A summary of lessons learned to date and future priorities

**DOI:** 10.7189/jogh.04.020303

**Published:** 2014-12

**Authors:** Mark Young, Alyssa Sharkey, Samira Aboubaker, Dyness Kasungami, Eric Swedberg, Kerry Ross

**Affiliations:** 1 UNICEF, New York, NY, USA; 2 World Health Organisation, Geneva, Switzerland; 3 Maternal and Child Survival Program (MCSP)/John Snow International, Washington, DC, USA; 4 Save the Children, Fairfield, CT, USA; 5 USAID, Washington, DC, USA

## SUMMARY OF “LESSONS LEARNED” FOR ICCM PROGRAMMING

Integrated community case management (iCCM) programming is an important and increasingly common strategy used to deliver essential health and nutrition interventions to families in sub–Saharan Africa [1–3]. Between 3 and 5 March 2014, over 400 individuals from 35 countries in sub–Saharan Africa and 59 international partner organisations gathered in Accra, Ghana for an iCCM Evidence Review Symposium. The objective of the Symposium was twofold: first, to review the current state of the art of iCCM implementation by bringing together researchers, donors, government, implementers and partners to review the map of the current landscape and status of evidence in key iCCM programme areas, in order to draw out priorities, lessons and gaps for improving child and maternal–newborn health and nutrition. Second, to assist African countries to integrate and take action on key frontline iCCM findings presented during the evidence Symposium around eight thematic areas: 1) Coordination, Policy Setting and Scale up; 2) Human Resources and Deployment; 3) Supervision & Performance Quality Assurance; 4) Supply Chain Management; 5) Costs, and cost-effectiveness and financing; 6) Monitoring, Evaluation and Health Information Systems; 7) Demand generation and social mobilisation; and 8) Impact and outcome evaluations. The eight thematic areas were based on the CCM benchmark framework, a tool for iCCM program planners and managers to systematically design and implement iCCM programs from the early phases through expansion and scale up. The framework specifies key steps that should be completed for each component and phase of implementation [4].

The evidence presented at the 2014 iCCM Symposium and included in this journal theme issue illustrates the tremendous progress made in iCCM programming in recent years and the range of innovations that have facilitated this progress across the thematic areas. Some examples of practices that have been effective and should now be scaled up include improvements in data availability and quality (as well as new tools to collect and disseminate data) [5–7] which have helped in critical areas such as costing and monitoring [8], investments in demand generation activities which have improved utilisation of services [9] and participatory, targeted, and adult–focused training methodologies which have been used successfully in some settings to improve the quality of care provided [10,11].

This collection of papers also sheds light on the numerous challenges that remain. For example, engaging “champions” of iCCM (at policy and technical levels) has been difficult in many countries in sub–Saharan Africa [12]. Similarly, the collection and use of routine data for programming as well as collection of financing data on iCCM programmes [5,7,8], ensuring continuous medicine and supplies [6,13], and retention and motivation of trained and remunerated community–based health workers have been challenging [10,11,14].

## KEY MESSAGES OF THE SYMPOSIUM

Two overall messages emerged from the Symposium evidence review and discussions with country implementers.

1. Increase utilisation of iCCM

First, in order to be cost–efficient and to ensure maximum impact of efforts it is critical to increase utilisation of iCCM. This can be supported by:

• deploying community health workers (CHWs) to areas of greatest need,

• assessing demand barriers and addressing them through behaviour change, community engagement and social mobilisation activities,

• ensuring continuous supplies, while at the same time maintaining quality of services and

• structuring supervision and management to be affordable and effective.

2. Monitoring and evaluation must be appropriate

Second, routine reporting data should be used to assess and monitor programme progress and endline impact evaluations should only be conducted after an iCCM programme has been operating at scale (that is, when 80 percent of providers have been trained and equipped) with high utilisation for at least one year. Specific steps recommended to ensure appropriate monitoring and evaluation include: 1) examine routine data to determine if high rates of appropriate treatments are being provided, 2) once routine data show high rates of treatment are being provided, collect data on coverage and quality, and model mortality based on the Lives Saved Tool (LiST), and 3) use data from routine sources, as well as contextual, qualitative, coverage, quality of care and costing data to conduct final evaluations.

In addition, operational research should be conducted as a priority over impact evaluations in order to determine how programmes can increase their coverage rates, effectiveness and cost–effectiveness. Utilising household surveys (baseline and follow up) to measure care seeking behaviour, source of treatment and timeliness of treatment to assess if these outcomes are moving in the right direction is also critical.

There were several clear “lessons learned” shared at the Symposium based on experiences to date. At the closing of the Symposium, these were presented as recommendations to improve the success of future iCCM implementation (**Box 1**).

Box 1 **Recommendations to improve iCCM programme implementation, as presented at the closing of the Evidence Review Symposium**

• National government leadership is essential.

• iCCM must be integrated in national health systems and seen as a priority means of delivering care, and embedded as a costed element of national health sector plans, with a clear budget line.

• Integration and collaboration is key among all health–related programmes at community level (water and sanitation, nutrition, etc.).

• Coordination mechanisms should extend beyond health to include other sectors (eg, finance).

• High levels of utilisation are necessary for iCCM programs to be cost–efficient and have maximum impact. Utilisation is supported by deploying staff to areas of greatest need, assessing and addressing demand barriers, ensuring continuous supplies, maintaining quality of services and structuring supervision and management to be affordable and effective.

• Providing integrated treatment for malaria, pneumonia and diarrhoea increases utilisation of services for each illness.

• Using rapid diagnostic tests (RDTs) promotes appropriate treatment of malaria and pneumonia suggesting more appropriate antibiotic/antimalarial usage and improved quality of treatment.

• Private public partnerships should be explored as vehicles for iCCM implementation. In addition, iCCM can be used as vehicle for private sector quality improvement in settings where the private sector is an important source of care for children.

• New mHealth technologies such as *Rapid SMS, cStock*, and *mTRAC* can facilitate monitoring and management.

• iCCM programmes must be well documented, periodically reviewed and evaluated in order to guide implementation at scale.

## KEY PRIORITIES IDENTIFIED IN COUNTRY ACTION PLANS

In order to support ministries of health to address relevant challenges in their local contexts, a major objective of the March 2014 iCCM Evidence Review Symposium was to assist African countries to integrate and take action on key iCCM findings relating to the eight thematic areas of the Symposium.

Country work had three phases: 1) pre–Symposium, to analyse and summarize the status of iCCM programs, including major achievements and challenges. 2) At the symposium, based on the evidence presented, country teams (which were comprised of government, UNICEF, WHO and other partner staff) identified priority actions to be addressed in 6 and 12 months respectively. To help countries during the Symposium, templates addressing each thematic area, were developed and used to record key messages coming out of each presentation or discussion. 3) Finally, country teams reviewed the key messages and identified those that they felt were relevant to their contexts. These were translated into final country specific action plans. Out of 35 countries from Sub–Sahara Africa, 26 submitted action actions. Post–Symposium, the teams were expected to share the action plans with a wider group of in country stakeholders, adjust as necessary and incorporate a final list of actions in existing iCCM workplans. After the Symposium, country priorities were summarised in a spreadsheet (2 × 2 table of thematic area and country) by all eight thematic areas against 26 countries. This formed the basis for the actions under each thematic area described in **Boxes 2**** and ****3**.

Box 2 **Common key activities from country action plans relating to coordination and policy setting, supply chain management, and human resources and deployment**

**Coordination and policy setting**

1. Adoption/expansion of policies that would support integration of iCCM into national policies (eg, community health policy, child survival policy, community health worker policy, integration of iCCM in national strategic plans, improving tools for iCCM, ensuring that iCCM policies and implementation are reflective of one another).

2. Adoption/expansion of policies to include improved access of CHWs to essential commodities (eg, amoxicillin, zinc, oral rehydration salts, and RDTs).

3. Inclusion of newborn care into existing community health/child health/iCCM policy.

4. Improved inter– and intra–sectoral coordination and collaboration between iCCM programmes and other child health related programmes (eg, between iCCM implementers and national government ministries including health and finance vis–ŕ–vis sensitisation, between district councils, among NGOs, formation of task forces, formation of working groups, establishment of knowledge–sharing mechanisms).

**Supply chain management**

1. Decrease of drug stockouts (by ensuring that stocking of drugs is based on *real*, rather than *perceived* needs and collecting data to determine drug needs and commodity gaps).

2. Exploration of innovations to improve supply chain efficiency (eg, mHealth for more accurate data collection; performance–based supply chains).

**Human resources and deployment**

1. More strategic methods for recruitment (through mapping of CHWs to determine geographic areas for increased coverage, selection of the most appropriate people to fill iCCM CHW role)

2. Improved quality and frequency of CHW trainings (before and during deployment, revision of tools to be the most appropriate for the specific cadre’s needs (eg, job aids, mentoring)

3. Exploration of incentivizing/motivating CHWs (eg, performance–based incentives, value in moving from volunteer to paid cadre, Living Goods’ micro–franchise model).

Box 3 **Priority actions identified by country teams participating in the iCCM Symposium**

**Monitoring and evaluation**

• introducing/strengthening HMIS at community level

• improving/standardizing monitoring

• improving the use of indicators and data collection methods

**Supervision and performance quality assurance**

• standardizing/strengthening supervision of CHWs

• starting to collect (or improving collection of) data on treatment, referral, and supplies, etc.

• developing better tools to support CHW supervisors

• exploring reporting incentives and the peer supervision model

**Costing, cost–effectiveness, and financing**

• increasing government funding for an integrated package of services

• using gap analyses and studies to identify iCCM programme costs

**Demand generation and social mobilisation**

• building the capacity of CHWs to utilize behaviour change communication strategies

• increasing demand for iCCM services by ensuring consistent access to commodities

**Other priority actions**

• identifying potential private sector partnerships (through strengthened coordination with those that already exist

• building private sector capacity through IMCI training and local commodities production

• integrating/scaling–up newborn care in iCCM services provided by CHWs

• integrating mHealth into iCCM programmes for a range of functions such as planning, logistics, monitoring and supervision, improved data management and reporting, and well as a method for paying CHWs.

While there were important differences across the stated priorities and action plans of the diverse countries in attendance at the Symposium, in part influenced by the current stage of iCCM programming, some summary points can be informative. Themes that resonated across country teams included coordination and policy setting, supply chain management, and human resources and deployment. Specific actions relating to country themes that were identified are listed in **Box 2**.

Priority actions were identified by some country teams for the other thematic areas as well (**Box 3**).

Once each country identified their priority activities, country teams worked together to discuss the key financial and human resources, and technical support needed to support their identified priority areas of work.

Although countries identified many problems related to iCCM programming that are well known, the Symposium provided them with opportunities for sharing best practices and lessons learned as well opportunities for learning about innovative solutions that countries are using to solve traditional problems. The messages shared by some of the country level senior decision makers in attendance (Niger, Ethiopia) also provided extra motivation for countries as they were able to see that with limited resources but with the right leadership and commitment, it is possible to make significant progress in reducing under five mortality.

## REMAINING RESEARCH QUESTIONS

While much information was shared regarding lessons learned about successful policy development, programme implementation and monitoring and evaluation of iCCM programmes, several critical questions remain. In 2013, the CCM Operational Research Group (CCM.ORG) led a process to identify research priorities for iCCM over the next five to ten years. These were presented at the Symposium. With the support of the Centre for Global Child Health at the University of Toronto, the process was guided by the Child Health and Nutrition Research Initiative (CHNRI) method [15]. The conclusions and recommendations from the evidence review re–enforce the priority research questions identified by the CHNRI process [16], with the former identifying the gap or needed action (for example, the need to increase utilization of services) and the later addressing the priority research questions that could facilitate increased utilization of services (for example, which strategies best ensure continuous availability of drugs, what are the best ways to generate demand??). The conclusions from the evidence review also provided additional topics for research that may have not been identified or didn’t rank highly in the CHNRI scoring. For example: What are successful models of integrating iCCM and financing in health sector plans and national budgets? The paper focusing on the CHNRI approach used to identify priority research questions for iCCM includes more information about the methods and process used [16]. As indicated in **Box 4**, strategies to improve motivation, retention, training and supervision of CHWs ranked highly among global and country experts, as did strategies to increase uptake of iCCM through demand generation, identification of determinants of non–use, motivating factors for care–seeking behaviour and improving compliance.

Box 4 **Top 10 iCCM research priorities identified through 2013 global CHNRI exercise**

1. Assess perceptions of beneficiaries and levels of community satisfaction in CHWs’ capacity to diagnose and treat sick children (with malaria, pneumonia, diarrhoea and severe malnutrition) at the community level.

2. Identify and evaluate strategies for retention and motivation of CHWs.

3. Identify and evaluate strategies for improving referral between communities and health facilities, including referral compliance.

4. Identify determinants of non–use of iCCM services by caretakers and develop strategies to increase the uptake of iCCM.

5. Identify and evaluate new diagnostic tools for improved classification of pneumonia (ie, different ARI timers, respiratory counting beads, etc.) at the community level that are most appropriate for various cadres.

6. Evaluate the effectiveness of 3–day vs 5–day oral amoxicillin treatment in Africa.

7. Identify and evaluate innovative strategies to improve community engagement and mobilisation for CCM.

8. Evaluate the feasibility, effectiveness and impact of adding community–based infant and young child feeding (cIYCF) counselling skills to the CHW workload.

9. Identify the primary barriers to CHW supervision and develop and evaluate strategies to motivate CHW supervisors to provide continuous support to CHWs.

10. What is the impact of pre–referral antibiotics on treatment outcomes of possible serious bacterial infections?

## CONCLUSION

The time for improving iCCM implementation is now. As illustrated by the papers included in this theme issue, we have effective interventions that address the major causes of child mortality and are well packaged for delivery. We have evidence showing that many treatments can be delivered successfully in the community, and although many problems implementing these interventions are not new, we now have innovations that facilitate community–based programming, including rapid diagnostic tests (RDTs) and mobile technologies, as well as improved and user–friendly medicines. In addition, there are important new opportunities to mobilise resources from domestic as well as external funds (eg, the Global Fund to Fight AIDS, Tuberculosis and Malaria, the World Bank’s Health Results Innovation Trust Fund/Results Based Financing initiative) and, in many countries, to integrate public private partnerships with government systems. In particular, by the end of 2014, it is expected that more than a dozen countries will submit concept notes with iCCM components to the Global Fund. A key challenge for these countries will be identifying the necessary co–financing opportunities from government and partners to complement Global Fund inputs for comprehensive iCCM programme scale up.

**Figure:**
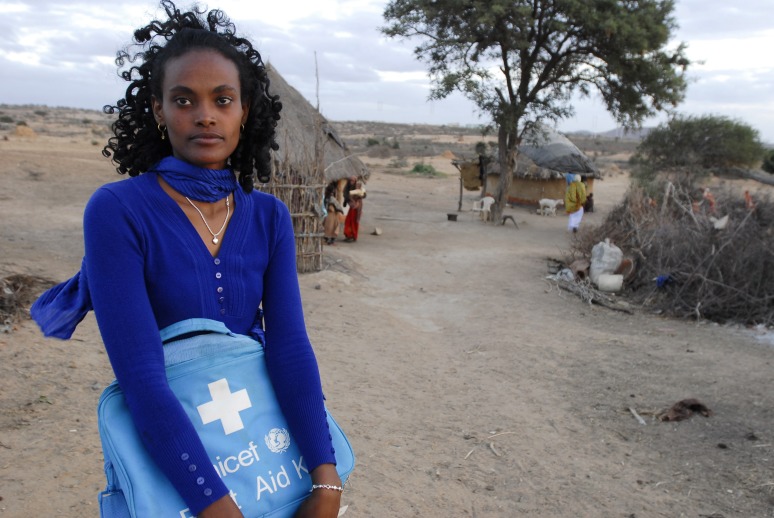
Photo: Courtesy of Indrias Getachew, UNICEF (© UNICEF/INDRIAS GETACHEW)

All of these activities can be supported with the advocacy platforms provided through stated country commitments for A Promise Renewed (APR), Every Woman Every Child (EWEC) and USAID’s Ending Preventable Child and Maternal Deaths (EPCMD) vision. Critical priorities should include strengthening and supporting country coordination, policy setting and leadership (perhaps through the identification of “iCCM champions” in ministries of health) as well as expanding iCCM programmes to scale. Acting on identified barriers relating to supply chain management and human resources and deployment may be particularly critical, as these appear to be key challenges across many settings in sub–Saharan Africa.

Following discussions about the opportunities and challenges in their respective countries, participants are poised to work with their colleagues and partners to ensure that iCCM programmes are based on the latest evidence and are most appropriate for, and integrated into, their particular health systems and contexts.
